# Good practices for the translation, cultural adaptation, and linguistic validation of clinician-reported outcome, observer-reported outcome, and performance outcome measures

**DOI:** 10.1186/s41687-020-00248-z

**Published:** 2020-11-04

**Authors:** Shawn McKown, Catherine Acquadro, Caroline Anfray, Benjamin Arnold, Sonya Eremenco, Christelle Giroudet, Mona Martin, Dana Weiss

**Affiliations:** 1 RWS Life Sciences, East Hartford, CT USA; 2 ICON plc, Patient Centred Sciences, Lyon, France; 3 Mapi Research Trust, Lyon, France; 4 FACITtrans, Ponte Vedra, FL USA; 5 Critical Path Institute, Tucson, AZ USA; 6 ICON plc, Language Services, Dublin, Ireland; 7 Evidera, Bethesda, MD USA; 8 Chicago, Illinois USA

**Keywords:** Translation, Linguistic validation, Clinical outcome assessments, Clinician-reported outcome measures, Observer-reported outcome measures, Performance outcome measures

## Abstract

Within current literature and practice, the category of patient-reported outcome (PRO) measures has been expanded into the broader category of clinical outcome assessments (COAs), which includes the subcategory of PRO, as well as clinician-reported outcome (ClinRO), observer-reported outcome (ObsRO), and performance outcome (PerfO) measure subcategories. However, despite this conceptual expansion, recommendations associated with translation, cultural adaptation, and linguistic validation of COAs remain focused on PRO measures, which has created a gap in specific process recommendations for the remaining types. This lack of recommendations has led to inconsistent approaches being implemented, leading to uncertainty in the scientific community regarding suitable methods. To address this gap, the ISOQOL Translation and Cultural Adaptation Special Interest Group (TCA-SIG) has developed recommendations specific to each of the three COA types currently lacking such documentation to support a standardized approach to their translation, cultural adaptation, and linguistic validation. The recommended process utilized to translate ObsRO, ClinRO and PerfO measures from one language to another aligns closely with the industry standard process for PRO measures. The substantial differences between respondent categories across COA types require targeted approaches to the cognitive interviewing procedures utilized within the linguistic validation process, including the use of patients for patient-facing text in ClinRO measures, and the need to interview the targeted observers for ObsROs measures.

## Background

Clinical outcome assessments (COAs), defined by the United States Food and Drug Administration (FDA) as tools that “measure a patient’s symptoms, overall mental state, or the effects of a disease or condition on how the patient functions,” are widely utilized within global clinical trials as a means of assessing concepts of interest and determining whether clinical benefit has been demonstrated [1]. COAs are categorized into four types: patient-reported outcome (PRO), clinician-reported outcome (ClinRO), observer-reported outcome (ObsRO), and performance outcome (PerfO) measures [1]. While use of these classifications has become widespread, it is also relatively new. Previously, regulatory bodies and industry users referred primarily to PRO measures rather than the broader category of COAs. This approach was favored throughout the literature, most notably in FDA’s *Guidance for Industry on Patient-Reported Outcome Measures* from December 2009, which specifically addressed PRO translation methodology guidance [2].

Utilization of the broader COA concept, encouraging readers to consider PRO measures as one of several COA types rather than as the primary focus, likely entered the dialogue in 2013 with the FDA’s release of the COA Roadmap to Patient-Focused Outcome Measurement [3]. This roadmap encouraged clinical trial personnel to select from the four COA types noted above to measure clinical benefit in treatment trials. In 2014, FDA released the Qualification Process for Drug Development Tools, which further developed this shift and featured guidance for COA qualification, encouraging users to select specific COA types as part of the trial planning process [4]. In 2018, FDA expanded this approach by releasing a Patient-Focused Drug Development (PFDD) draft guidance which highlighted recommended processes for selecting, developing or modifying fit-for-purpose COAs [5].

As use of the preferred concept has widened from PRO to COA within industry and literature, a gap in recommendations associated with translation, cultural adaptation, and linguistic validation processes has developed. The robust and effective guidance developed by FDA in 2009 [2], as well as the International Society for Pharmacoeconomics and Outcomes Research (ISPOR) *Principles of Good Practice for the Translation and Cultural Adaptation Process for Patient-Reported Outcomes (PRO) Measures* published in 2005 [6], apply specifically to PRO measures, and do not explicitly address the procedural requirements for the development, cultural adaptation, and/or linguistic validation of ObsRO, ClinRO, or PerfO measures. Translation service providers and academic groups performing cultural adaptations or linguistic validation currently do not have access to consensus recommendations specific to these COA types, leading to inconsistent approaches across these stakeholders.

While it is becoming more common for all COAs being used in clinical trials to be translated, there are still some cases where pharmaceutical sponsors elect not to translate these COA types, particularly ClinRO measures. This may, in part, be due to the lack of current guidance and in part due to an assumption that clinicians and staff personnel will speak English well enough to complete the translations adequately. Both issues are of concern because it opens the door for lack of consistency in the interpretation and presentation of the data collection as all users, with varying language abilities, are expected to produce the same concept equivalency.

Lack of existing guidance for ClinRO, ObsRO, and PerfO measures is of particular concern from a methodological perspective because the existing PRO process recommendations include “cognitive debriefing of the new translation, usually with patients drawn from the target population,” a recommendation that cannot directly apply to all of the COA types due to its requirement of individual cognitive debriefing interviews with patients, as opposed to observers or clinicians [6]. This is true and can seem obvious for the cognitive debriefing step, but one can also question whether there might be other aspects specific to non-PRO COAs that should be addressed specifically from a cultural and conceptual perspective. In order to address this gap, the ISOQOL Translation and Cultural Adaptation Special Interest Group (TCA-SIG) has developed recommendations specific to each of the three COA types which currently lack such documentation through a consensus approach. These recommendations are designed to align process expectations across stakeholders and address the existing gap in process good practices.

### Recommendations for ObsRO, ClinRO and PerfO measures

To understand this broader COA concept which has replaced the PRO concept in recent years, it is important to identify distinctions between the COA types. Table 1 presents definitions for each non-PRO COA type.

**Table 1:** Definitions of non-PRO COA types

COA type	National Center for Biotechnology Information (NCBI) Definition
Observer-reported outcome measure (ObsRO)	A measurement based on a report of observable signs, events or behaviors related to a patient’s health condition by someone other than the patient or a health professional. Generally, ObsROs are reported by a parent, caregiver, or someone who observes the patient in daily life and are particularly useful for patients who cannot report for themselves (e.g., infants or individuals who are cognitively impaired). An ObsRO measure does not include medical judgment or interpretation [7].
Clinician-reported outcome measure (ClinRO)	A measurement based on a report that comes from a trained health-care professional after observation of a patient’s health condition. Most ClinRO measures involve a clinical judgment or interpretation of the observable signs, behaviors, or other manifestations related to a disease or condition. ClinRO measures cannot directly assess symptoms that are known only to the patient [7].
Performance outcome measure (PerfO)	A measurement based on standardized task(s) performed by a patient that is administered and evaluated by an appropriately trained individual or is independently completed [7].

Proxy measures are excluded from the ObsRO measures category because these measures require that an informant report as if he or she was the patient. The FDA notes that “for patients who cannot respond for themselves (e.g., infants or cognitively impaired), we encourage observer reports that include only those events or behaviors that can be observed. As an example, observers cannot validly report an infant’s pain intensity (a symptom) but can report infant behavior thought to be caused by pain (e.g., crying)” [1]. COAs intended for completion by caregivers which collect information about the caregiver’s personal feelings and experiences are similarly excluded from the ObsRO measure category.

While specific recommendations for their translation are currently lacking, uses of ObsRO, ClinRO and PerfO measures in clinical trials have been presented in literature, workshops, and studies by task forces within ISPOR. An ISPOR Task Force reviewed use of PRO and ObsRO measures in rare disease trials and produced an emerging good practices report, noting that “further incorporation of the patient-perspective requires the inclusion of PROs for patients who can speak for themselves … [and] ObsROs by parents and caregivers for those who cannot” [8]. A 2017 article by Powers and colleagues focused on issues related to development and evaluation of ClinRO measures in evaluating treatment benefit [9]. Increasing focus on these COAs within the multinational clinical trial space, particularly for pediatric, rare disease, and cognitively impaired populations, indicates a need to develop distinct and rigorous methodology recommendations for their translation, cultural adaptation, and linguistic validation.

## Methods

### Authorship

Authorship was determined on a volunteer basis from the pool of 130 ISOQOL TCA-SIG members. Volunteers were solicited for lead and contributing author roles based on COA type, with a different lead ultimately volunteering for each of the three non-PRO COA types (ObsRO, ClinRO, PerfO). A literature review group was also convened. These working groups consisted of representatives from non-profit (Critical Path Institute, Mapi Research Trust), academia (University of Washington), pharmaceutical industry (Janssen), and companies specializing in translation (Amplexor, FACITtrans, HRA/Evidera, ICON/Mapi, RWS Life Sciences), all with significant experience in reviewing and translating ObsRO, ClinRO, and PerfO measures.

### Literature review

A sub-group was convened to identify publications which had previously explored the use of ObsRO, ClinRO, and PerfO measures in clinical trials, with particular attention paid to cross-cultural use and translation methodology of these measures. Results were compiled, consolidated, and provided to the methods working group for further discussion.

### Creation and distribution of methodology questionnaires

Three questionnaires were designed to collect information regarding ObsRO, ClinRO, and PerfO measure translation methodology among experts in the field (Additional file 1: Appendices A, B, and C in the online supplement). These questionnaires were administered online in English and contained between 15 and 19 items that were developed and refined by the working groups. Items largely focused on process specifics, as well as any elements that could distinguish the ObsRO, ClinRO or PerfO measure translation processes from the more well-documented processes utilized for PRO translation and linguistic validation. Items asked about frequency of projects, methodology differences compared to standard PRO project methodology, process steps required for translation and cognitive interviews/pilot testing, and process considerations specific to ObsRO, ClinRO and PerfO measures. Questionnaires were designed to include questions specific to their COA type, such as a question about observer categories in the ObsRO questionnaire, a question about clinician input in the ClinRO questionnaire, and questions about engaging with cognitively impaired patients in the PerfO questionnaire. The intent of the questionnaires was to gather insight into current practices and to identify potential best practices for consideration by the writing team. The team looked to see where there seemed to be consensus among the respondents and where there were areas of disagreement. Areas of consensus were discussed as a group to ensure agreement with the recommended best practice. For areas of disagreement, the team discussed and worked to achieve consensus, taking the survey results into consideration.

The ObsRO and ClinRO questionnaires were distributed to a total of 27 individuals representing 27 organizations, while the PerfO questionnaire was distributed to 35 individuals representing 34 organizations. Although the content of the questionnaires was targeted specifically to the translation process, a variety of organizations were invited to participate, including representatives from translation companies, COA developers, pharmaceutical sponsors, academia, non-profit, government, electronic COA (eCOA) vendors, and contract research organizations (CROs). The questionnaires were completed in an online survey between August 2017 and October 2017.

## Results

### Overview of questionnaire results

Questionnaire responses were received from representatives of 10 organizations (Amplexor, Critical Path Institute, Signant Health, FACITtrans, HRA/Evidera, Lionbridge, ICON/Mapi, Oxford University Innovation, RWS Life Sciences, and TransPerfect). These organizations represent a good cross-section of experts in the field with decades of global, cross-cultural COA and linguistic validation expertise and experience. Each individual respondent was asked to complete three questionnaires (48 items total). Respondents were given the ability to skip questions according to their preferences and areas of expertise, which led to varying denominators per item during analysis. Respondents included representatives from translation companies, instrument developers, eCOA companies, and non-profit organizations. Two additional respondents completed some but not all of the questionnaires, and their organizational data was not captured as a result. As the surveys were completed anonymously, the ethnicities and countries of residence of the respondents are unknown because this information was not collected as part of the survey. The organizations of the respondents are headquartered in France, Ireland, the United Kingdom, and the United States.

The results indicated broad agreement among respondents regarding general experiences with, and approaches to, the linguistic validation of COAs. Most (27/33; 82%) responses indicated that requests for ObsRO/ClinRO/PerfO measure translation projects were either less common (18/33; 56%) or much less common (9/33; 27%) than requests for PRO measure translation projects. Most (25/33; 76%) responses indicated that ObsRO/ClinRO/PerfO measure translation projects usually take the same amount of time to set up as PRO measure translation projects.

Respondents also reported broad agreement regarding translation and linguistic validation methodology for COA projects. The following translation process steps were recommended by over 70% of responses:

- Creation of concept definition document (28/30; 93%)
- Developer review of concept definition document (28/30; 93%)
- Dual forward translations (28/30; 93%)
- Reconciliation of forward translations (27/30; 90%)
- Single back-translation (24/30; 80%)
- Project Manager review and evaluation of back-translation (29/30; 97%)
- Developer review of back-translation evaluation (22/30; 73%)
- Proofreading (27/30; 90%)

The most substantial translation process step difference between the COA type responses related to the issue of in-country clinician review of the translation. While the vast majority of respondents indicated that clinician review was necessary from the ClinRO (9/11; 82%) and PerfO (9/10; 90%) groups, a clinician review was not deemed necessary by the respondents from the ObsRO group (2/9; 22%).

Responses to the ClinRO questionnaire did diverge from the ObsRO and PerfO questionnaires in less substantial ways in terms of translator guidance provided and overall project length. While most (14/20; 70%) responses to the ObsRO and PerfO questionnaires indicated that there are no differences in the guidance they provide to translators as compared to PRO projects, few respondents of the ClinRO questionnaire agreed (3/11; 27%). Similarly, while most (15/21; 71%) responses to the ObsRO and PerfO questionnaires indicated that there are no differences in length of translation projects as compared to PRO projects, ClinRO respondents indicated that translation projects were shorter than PRO projects (8/12; 67%).

### Cognitive interviewing (pilot testing)

In contrast to the relative agreement on translation methodology observed across respondent groups, review of the preferred cognitive interviewing process elicited unique and distinct methodology recommendations from each group.

### Cognitive interviewing (pilot testing): ObsRO measures

The following cognitive interview/pilot testing process steps were recommended by over 70% of respondents to the ObsRO questionnaire:

- Cognitive interviews with the patients’ caregivers (as applicable) (8/9; 89%)
- Cognitive interviews with other observers of the patient (as applicable) (7/9; 78%)
- For adult patients, interviews should be completed in-person with observer, with the patient not in the room (9/9; 100%)
- For pediatric patients, interviews should be completed in-person with observer, with the child not in the room (8/9; 89%)

Questionnaire results uncovered some areas of disagreement related to specific challenges presented by the cognitive interviewing of translated ObsRO measures. When queried about whether a restriction should be placed on the maximum amount of time since the observer respondent last observed the patient’s behavior, the responses were split (55% [5/9] favored no restriction, 45% [4/9] favored including a restriction). For those respondents that favored a restriction, there was no consensus on what the restriction should be, and responses ranged from 1 week to 6 months. There was similarly no consensus on the question of how to approach cognitive interviews for ObsRO measures which indicate more than one observer type (i.e., parent, caregiver, teacher). Issues which did not show clear consensus within the questionnaire results were referred to the working group for further discussion and resolution.

### Cognitive interviewing (pilot testing): ClinRO measures

No specific cognitive interview/pilot testing process steps were recommended by over 70% of respondents to the ClinRO questionnaire. Six of nine respondents indicated that cognitive interviews with patients should be undertaken in cases where the ClinRO measure contains patient-facing text. Five of nine respondents expressed a preference for including cognitive interviews with clinicians, while other respondents described interviewing clinicians as being less effective than a clinician review of the text.

### Cognitive interviewing (pilot testing): PerfO measures

The following cognitive interview/pilot testing process steps were recommended by over 70% of respondents to the PerfO questionnaire:

- Pilot testing with patients should be performed (administering the PerfO measure where respondents will perform the tasks): (7/10; 70%)
- Cognitive interviews with patients should be performed, where patient-facing parts (e.g., instructions, stimuli) are reviewed: (8/10; 80%)
- The PerfO tasks should be administered by the interviewer (5/7; 71%)
- Cognitive interviews with clinicians/healthcare professionals are **not** required (6/8; 75%)

Areas of weaker consensus regarding the PerfO cognitive interview process included:

- Whether cognitive interviews should be completed with the individual who administered the PerfO measure during pilot testing (60% [6/10] indicated “No”)
- Whether cognitively impaired patients should participate in cognitive interviews/pilot testing for measures intended for use with a cognitively impaired population (63% [5/8] indicated “Yes”).

## Discussion

While the regulatory and industry view of clinical outcomes measurement has shifted from focusing on PRO measures to focusing on the broader category of COAs, specific methodological guidelines regarding the translation, cultural adaptation, and linguistic validation of non-PRO COAs (i.e., ObsRO, ClinRO, and PerfO measures) does not currently exist. Our working group sought to develop clear, actionable, and achievable process recommendations to fill this gap and to align process expectations across stakeholders.

Our research found that the process utilized to translate ObsRO, ClinRO and PerfO measures from one language to another aligns closely with the process outlined in the ISPOR recommendations for translation and cultural adaptation of PRO measures. A summary of these recommended good practices for all COAs can be found in Table 2**.**

**Table 2:** Recommended COA Translation Process Steps

Step	Definition
Creation of concept definition document	The concept definition document, also known as a concept elaboration guide or an item definition document, contains information about the conceptual basis for each item or task in the measure. It is provided to all translators (except the back-translator) to ensure a consistent understanding of these concepts throughout the translation process.
Developer review of concept definition document	The original developer or those with the necessary scientific expertise regarding development of the COA should ideally participate in the creation or review of the concept definition document to ensure approval and alignment.
Dual forward translations	The source COA is provided to two separate professional linguists who are native speakers of the target language and have experience in translating COAs or other clinical trial documentation. Each linguist completes an independent translation into the target language, consulting the concept definition document as necessary during the process. Initial cultural adaptation should occur during the forward translation process and is particularly important to consider when translating PRO and PerfO measures. This translation process should be applied to ClinRO measures, which are too often not translated due to the assumption that clinicians and site personnel are sufficiently fluent in English.
Reconciliation of forward translations	Reconciliation of the dual forward translations into one translation. Per ISPOR recommendations, this can be completed either by the two forward translators working together, or alternately by a third qualified translator working independently [10].
Single back-translation	The reconciled translation is provided to a professional linguist to perform a back-translation into the source language. The back-translation is to be completed by a linguist who does not have access to the original source COA or concept definition document. The purpose of the back-translation is to provide a quality control step which is used to ensure that the reconciled translation is conceptually equivalent to the source text.
Project Manager review and evaluation of back-translation	The back-translation is reviewed against the source language by the Project Manager. Any conceptual discrepancies or other problematic items identified are presented to a translator for review and discussion. Updates are made to the reconciled translation as required.
Developer review of back-translation evaluation	The back-translation is reviewed against the source language by the COA developer or those with the necessary scientific expertise in development of the COA. Any conceptual discrepancies or other problematic items identified are presented to the Project Manager and translator for review and discussion. Updates are made to the reconciled translation as required.
Native-speaking clinician review of the translation	The translation is reviewed against the source language by a clinician who is a native speaker of the target language, and who specializes in the condition being studied. Recommended specifically for ClinRO and PerfO measures, although this may be helpful as an optional step when translating ObsRO or PRO measures. For ClinRO and PerfO measures, use of an in-country Key Opinion Leader as the reviewer will serve to further improve acceptability of the version in the country and will also contribute to inter-rater reliability.
Proofreading	The reconciled translation is proofread by a linguist in preparation for cognitive interview/pilot testing activities.

While the translation process for these measures did not require substantial modification from the generally accepted PRO translation methodology, we found it of particular importance to highlight the necessity of translating all assessment material, including the components given to the clinician or the rater. This is especially true for cognitive assessments that are used to measure a person’s cognitive functioning. They are generally composed of 3 elements: (1) the Stimuli, (2) the Instructions to the patients (i.e. read by the rater), (3) the Instructions to the rater on how to administer and score the test.

The stimuli may include images, numbers, letters, words, short stories, objects from daily life, etc. The latter—that are presented to the patients—may need to be adapted to the country of interest, following the standards of cultural adaptation of patient-facing attributes. Particular attention will also need to be given to the material that is for the raters, i.e. the Response Form for the rater to write down the patient’s score (sometimes also containing the stimuli) and the Instruction Manual containing the instructions to the rater and instructions to the patient. For constraints of timelines or budget, this material to the rater is often neglected, poorly translated, or even not translated at all due to the assumption that clinicians and site personnel are sufficiently fluent in English. Expecting clinicians and site personnel to use English forms and instruction manuals can dramatically result in bias or incorrect interpretations of the measure content that threaten the validity of the data. To tackle this risk, performing a rigorous translation of the rater material will standardize the measure across all clinicians in a given country and between countries, which will improve inter-rater reliability of the translated measure itself.

Our research also found that the recommended cognitive interviewing/pilot testing process differs substantially between COA types. A summary of the recommended COA interview processes in comparison with the generally accepted PRO process can be found in Table 3.

**Table 3:** Cognitive Interview Recommendations by COA type

Process	Patient-reported outcome measure (PRO)	Observer-reported outcome measure (ObsRO)	Performance outcome measure (PerfO)	Clinician-reported outcome measure (ClinRO)
Performing individual interviews with subjects to complete item-by-item cognitive debriefing of the COA translation	Yes	Yes	Yes (for patient-facing text contained within the PerfO measure)	Yes (for patient-facing text contained within the ClinRO measure)
Interviewing in-country patients drawn from the target population	Yes	No	Yes (for patient-facing text contained within the PerfO measure)	Yes (for patient-facing text contained within the ClinRO measure)
Interviewing native-speaking clinicians who specialize in the condition being studied	No	No	No	An acceptable but not mandatory approach. Clinician review of the text is an acceptable alternative. Interviews with clinicians may be useful in cases where complexity and content of the ClinRO measure presents a vulnerability to varied interpretations.
Interviewing in-country observers of the patients drawn from the target population (patient not in the room during interview)	No	Yes	No	No
Selection of interview subjects based on observer type (parent, caregiver, teacher) required as well as disease population	No	Yes, although selection of observers may vary depending on the type most likely to be included in future trials and their availability for such interviews. Flexibility is recommended.	No	No
Pilot testing including patients performing specific tasks	No	No	Yes	No

### Further discussion: ObsRO measure cognitive interviewing

While both the questionnaire results and initial working group discussions revealed broad alignment on the procedural recommendations for ObsRO measures as noted above, there were areas of disagreement that were referred to the working group for further discussion.

The first problematic issue related to the definition of “observer,” and specifically whether a restriction should be placed on the maximum amount of time since the observer respondent last observed the patient’s behavior. After discussion and review, there was consensus that there should be some sort of restriction, as the memories of the observers would become unreliable over time. Once agreeing to the existence of a restriction, the group debated its length. A short window of time, such as days or weeks, was thought to exclude too many potential observers who would otherwise provide useful data, while too long a window would present the same challenge as requiring no restriction at all. Ultimately, the group agreed to recommend that a restriction of 1 month since the respondent last observed the patient’s behavior should be implemented. Future research may be needed to confirm the feasibility and necessity of this recommendation.

The second problematic issue was the question of how to approach cognitive interviews for ObsRO measures which indicate more than one observer type (e.g., parent, caregiver, and/or teacher). Questionnaire results and initial discussions among the working group showed little consensus. After discussion and review, the group noted that while some ObsRO measures may be applicable to multiple observer categories, specific clinical trials would more likely target a particular category of observer based on the needs of the trial. In the interest of making the translation deliverable fit-for-purpose, it was agreed to recommend that groups performing translations should take into consideration the observer type that will be utilized in the clinical trial associated with the project when determining which observer type to interview. In cases where this information is unavailable or inapplicable, vendors should attempt to perform interviews with multiple types of observers when an ObsRO measure has multiple observer types indicated.

### Further discussion: ClinRO measure cognitive interviewing

The questionnaire results related to performing cognitive interviews with ClinRO measures were less clear than the results related to other COA types. Overall, there was consensus that interviews with patients should be undertaken for patient-facing text in cases where patient-facing text is included within the ClinRO measure. There was not consensus, however, regarding whether clinicians themselves should be interviewed as part of the process. Ultimately, the working group decided to present clinician interviews as an acceptable but not mandatory approach, which could be supplemented or replaced by a clinician review of the translation in most cases.

### Further discussion: PerfO measure cognitive interviewing

Relatively minor differences of opinion regarding the cognitive interviewing process for PerfO measures were reviewed by the working group. It was determined that while conducting additional cognitive interviewing with the individual who administered the PerfO measure during pilot testing could be interesting and fruitful, it was not a mandatory component of the process. The questionnaire results indicated a slight preference (5/8) for interviewing patients with mild cognitive impairment when testing PerfO measures intended for use with a cognitively impaired population. The working group agreed with this approach, while noting that recruitment of cognitively intact subjects with other specific criteria (e.g., within a specific age range) was a reasonable alternate approach in cases where interviewing patients with cognitive impairment was ineffective or otherwise not feasible.

### Limitations

This paper focused on good practice recommendations for translation, cultural adaptation, and linguistic validation of ObsRO, ClinRO, and PerfO measures. Translatability assessment was not addressed as it is a separate process conducted during instrument development that precedes the translation process outlined here [11]. The ISOQOL TCA-SIG has published emerging good practice recommendations for translatability assessment of PRO measures but did not have sufficient evidence to expand the recommendations to non-PRO COAs [11]. Although one would expect the process to align closely with that of PRO measures, the need for good practice recommendations for translatability assessment of non-PRO COAs remains to be addressed.

### Summary

In order to develop reasonable and actionable good practice recommendations for the translation, cultural adaptation, and linguistic validation of non-PRO COAs, the ISOQOL TCA-SIG examined the characteristics and requirements of each COA type by means of a literature review, completion of targeted questionnaires by industry experts, and group discussion and analysis. Our findings indicate that while recommended translation process steps generally align across all COA types (including PRO measures), the substantial differences between respondent categories across COA types require targeted approaches to the cognitive interviewing procedures utilized within the linguistic validation process. As a result, specific good practices and process recommendations have been developed for each non-PRO COA type, which will assist in further aligning procedures across service providers, COA instrument developers, and industry sponsors.

## Supplementary information

**Additional file 1.**

## Data Availability

Contained in Additional file 1: Appendix A.
