# Supplementary material for: Good practices for the translation, cultural adaptation, and linguistic validation of clinician-reported outcome, observer-reported outcome, and performance outcome measures
Source: J Patient Rep Outcomes. 2020 Nov 4;4:89. doi: 10.1186/s41687-020-00248-z (PMC7642163; doi:10.1186/s41687-020-00248-z)
Supplement: Supplementary file 1 — Additional file 1. [file 41687_2020_248_MOESM1_ESM.docx]

Online Supplement Material

**Appendix A**

**Identifying Views about Observer-Reported Outcomes (ObsRO)
Methodology in Translation Work Survey**

**Observer-reported outcomes (ObsRO):** a measurement based on direct observation by someone other than the patient or a healthcare professional. This other person can be a parent, spouse, teacher, or any other non-clinical caregiver who is able to regularly observe and report on specific aspects of the patient’s health or condition.

**Project Set Up**

1. When thinking about Observer Reported Outcome (ObsRO) translation projects in your work, how commonly are ObsRO measures asked for compared to Patient Reported Outcomes (PRO) measures?
   1. Much more common
   2. More common
   3. Equally common
   4. Less common
   5. Much less common
2. When you are involved in the setup of an ObsRO translation project, are there differences in the time required to set up these translation projects relative to PRO measures?
   1. Yes, ObsROs usually take more time to set up relative to PRO measures
   2. No, ObsROs usually take the same amount of time to set up as PRO measures
   3. Yes, ObsROs usually take less time to set up relative to PRO measures
3. What is the turnaround time for completion of translation projects involving ObsROs relative to PRO measures?
   1. ObsROs usually take more time to complete relative to PRO measures
   2. ObsROs usually take the same amount of time to complete as PRO measures
   3. ObsROs usually take less time to complete relative to PRO measures

**Translation and Validation Process**

1. What types of differences do you find in the amount of explanation provided for correctly rendering concepts that are being measured in ObsROs relative to PRO measures?
   1. Usually more conceptual explanation or elaboration is provided over PRO measures
   2. No differences noted between conceptual explanations for ObsROs and PRO measures
   3. Less conceptual explanation is usually provided for ObsROs compared to PRO measures
2. What steps do you feel should be included in the translation of ObsROs? (mark all that apply):
   1. Creation of concept definition document
   2. Developer review of concept definition document
   3. Single forward translation
   4. Dual forward translation
   5. Reconciliation of forward translations
   6. Single back translation
   7. Dual back translation
   8. Project Manager review and evaluation of back translations
   9. Developer review of back translation
   10. Client subsidiary review of translation (i.e. in-country review by a study physician or by the on-site field coordinator)
   11. Clinician review (not by client affiliate)
   12. Proofreading
   13. Other (Please specify:­­­­­­­­­­­­­­­­­­­­____________________________________________________)
3. Are there any differences in the guidance you provide to translators or reviewers regarding best practices for translation and linguistic validation when working with ObsRO measures vs PRO measures?
   1. Yes
   2. No

Please explain your answer in a sentence or two:

**Recruitment and Cognitive Debriefing Interview Differences**

1. When completing cognitive debriefing/pilot testing interviews for an ObsRO, please select all of the process steps you would generally include:
2. Cognitive debriefing interviews with patients
3. Cognitive debriefing interviews with the patients’ caregivers
4. Cognitive interviews with other observers of the patients
5. Cognitive debriefing interviews with “healthy” subjects (those without a particular disease or illness)
6. Administration of follow-up probe questions (developed internally)
7. Administration of follow-up probe questions (provided by developer or sponsor)
8. Review of cognitive debriefing methodology by developer or sponsor prior to interviews
9. Review of cognitive debriefing methodology data by developer or sponsor prior to translation delivery
10. Developer review of cognitive interview results
11. Which types of observers have you encountered in ObsRO questionnaires? (Please select all that apply.)
    1. Caregivers (unpaid)
    2. Caregivers (paid)
    3. Parents
    4. Spouses
    5. Teachers
    6. Other (Please specify:__________________________________________)
12. When an ObsRO has multiple possible types of observers indicated (e.g. a parent, sibling, OR spouse could answer the questionnaire), with whom do you perform cognitive debriefing interviews?
    1. Consistently use only one type of observer
    2. Use observers based on which type is most available
    3. Try to use all types of observers, but it’s not mandatory
    4. Mandatorily use all potential types of observers
13. When recruiting observers (parents, caregivers, etc.) for cognitive debriefing interviews involving ObsRO measures, do you place a restriction on the maximum amount of time since the respondent last observed the subject’s behavior?
    1. Yes
    2. No

10a. **If yes**, what is the maximum amount of time allowed since the respondent last observed the subject’s behavior?

# field

Dropdown box:

Weeks

Months

Years

1. Are there any special considerations you would recommend when interviewing parent and caregiver observers? (Please select all that apply.)

**Parent ObsROs**

- 1. In-person interviews, child **in** the room with parent
  2. In-person interviews, child **not in** the room with parent
  3. Telephone interviews, child **in** the room with parent
  4. Telephone interviews, child **not in** the room with parent
  5. No special considerations made

**Caregiver ObsROs**

- 1. In-person interviews, patient **in** the room with caregiver
  2. In-person interviews, patient **not in** the room with caregiver
  3. Telephone interviews, patient **in** the room with caregiver
  4. Telephone interviews, patient **not in** the room with caregiver
  5. No special considerations made

**Developer Involvement**

1. Are there any differences in the level of client or developer involvement in a project with ObsRO measures relative to PRO measures?
   1. **More** developer involvement relative to PRO measures
   2. **Equal** level of developer involvement relative to PRO measures
   3. **Less** developer involvement relative to PRO measures
2. If there are any other comments you would like to contribute relative to the translation process for ObsROs, please write those comments here.
3. Thank you for your participation. Please provide the name of the company or organization you represent below.

**APPENDIX B**

**Identifying Views about Clinician-Reported Outcomes (ClinROs)
Methodology in Translation Work Survey**

A **clinician-reported outcomes** assessment (ClinRO) is conducted and reported by a trained health care professional and requires specialized professional training to evaluate the patient’s health status. The primary feature of all ClinROs are that they call upon some aspect of clinical judgement.  There are three main types of ClinROs:  (1) Readings (usually dichotomous in nature, indicating the presence or absence of a feature or a state; (2) Ratings (which can be categorical or continuous but offer description of a patient’s state; and (3) Global impressions (which can be about a state or about change, but require an overall judgement from the clinician).

**Project Set Up**

1. When thinking about Clinician Reported Outcome (ClinRO) translation projects in your work, how commonly are ClinROs asked for compared to Patient Reported Outcomes (PRO) measures?
   1. Much more common
   2. More common
   3. Equally common
   4. Less common
   5. Much less common
2. When you are involved in the setup of a ClinRO translation project, are there differences in the time required to set up these translation projects relative to PRO measures?
   1. Yes, ClinROs usually take more time to set up relative to PRO measures
   2. No, ClinROs usually take the same amount of time to set up as PRO measures
   3. Yes, ClinROs usually take less time to set up relative to PRO measures
3. What is the turnaround time for completion of translation projects involving ClinROs relative to PRO measures?
   1. ClinROs usually take more time to complete relative to PRO measures
   2. ClinROs usually take the same amount of time to complete as PRO measures
   3. ClinROs usually take less time to complete relative to PRO measures

**Translation and Linguistic Validation Process**

1. What types of differences do you find in the amount of explanation provided for correctly rendering concepts that are being measured in ClinROs relative to PRO measures?
   1. Usually more conceptual explanation or elaboration is provided over PRO measures
   2. No differences noted between conceptual explanations for ClinROs and PRO measures
   3. Less conceptual explanation is usually provided for ClinROs compared to PRO measures
2. What steps do you feel should be included in the translation of ClinROs? (mark all that apply):
   1. Creation of concept definition document
   2. Developer review of concept definition document
   3. Single forward translation
   4. Dual forward translation
   5. Reconciliation of forward translations
   6. Single back translation
   7. Dual back translation
   8. Project Manager review and evaluation of back translations
   9. Developer review of back translation
   10. Client subsidiary review of translation (i.e. in-country review by a study physician or by the on-site field coordinator)
   11. Patient review (cognitive interview process) of patient facing aspects of a ClinRO
   12. In Country (in specialty) clinician review of translation (single clinician reviewer)
   13. In Country (in specialty) clinician review of translation (multiple clinicians)
   14. Developer review of cognitive interview results
   15. Proofreading
   16. Other (Please specify:­­­­­­­­­­­­­­­­­­­­____________________________________________________)
3. When completing translations of a ClinRO measure, what type of input from clinician(s) do you typically plan:
   1. Review of the translated material by clinician(s) – clinician(s) may propose(s) changes to translation but without direct discussion with project team
   2. Cognitive interview process with clinician(s) - specific questions asked to clinician(s) during interview about translated PerfO measure
   3. Another type of input from clinician(s), please describe: ____________________________________________________
   4. No clinician’s input planned
4. When completing translations of a ClinRO measure, do you typically plan input from clinician(s):
   1. At one point in the process
   2. At several points in the process. If so, please describe how many and when in the process: ____________________________________________________
   3. No clinician’s input planned
5. Are there any differences in the guidance you provide to translators or reviewers regarding best practices for translation when working with ClinRO measures compared to PRO measures?
   1. Yes
   2. No

Please explain your answer in a sentence or two:

1. Who should conduct the translation process for a ClinRO? (mark all that apply)
   1. Translation consultant (non-medical)
   2. Medical translator
   3. Clinical care professionals
   4. Other? (specify) _______________________________________

**Recruitment and Cognitive Interview Differences**

1. When completing cognitive interviews with ClinROs, what aspects would you include? (mark all that apply)
2. Cognitive interviews with clinicians (how many?) _____
3. Cognitive interviews with patients (how many?) _____
4. Review of cognitive interview methodology by developer or sponsor prior to interviews
5. Review of cognitive interview data by developer or sponsor prior to translation delivery
6. Please provide a short description of any differences you would suggest in translation methodology for the following types of ClinROs:
   1. Clinician Readings (for example of test materials):
   2. Clinician Ratings (of aspects of patient status):
   3. Clinician Global Ratings (of patient status):
   4. ClinROs that involve direct patient facing questions :
7. What is the general level of acceptable credentials for a reviewing clinician?
8. Are there any special considerations you would recommend for conducting cognitive interviews with clinicians?

**Developer Involvement**

1. Are there any differences in the level of client or developer involvement in a project with ClinRO measures relative to PRO measures?
   1. **More** developer involvement relative to PRO measures
   2. **Equal** level of developer involvement relative to PRO measures
   3. **Less** developer involvement relative to PRO measures
2. If there are any other comments you would like to contribute relative to the translation process for ClinROs, please write those comments here.

Thank you for your participation. Please provide the name of the company or organization you represent below.

**Appendix C**

**Identifying Views about Performance Outcomes (PerfO)
Methodology in Translation Work Survey**

**Performance outcome measure (PerfO):** a measurement based on a task(s) performed by a patient according to instructions that are administered by a healthcare professional. Performance outcomes require patient cooperation and motivation. These include measures of gait speed (e.g., timed 25 foot walk test), memory recall, or other cognitive testing (e.g., digit symbol substitution test).

**Project Set Up**

1. When thinking about PerfO measure translation projects in your work, how commonly are PerfO measures asked for compared to Patient-Reported Outcomes (PRO) measures?
   1. Much more common
   2. More common
   3. Equally common
   4. Less common
   5. Much less common
2. When you are involved in the setup of a PerfO translation project, are there differences in the time required to set up these translation projects relative to PRO measures?
   1. Yes, PerfOs usually take more time to set up relative to PRO measures
   2. No, PerfOs usually take the same amount of time to set up as PRO measures
   3. Yes, PerfOs usually take less time to set up relative to PRO measures
3. What is the turnaround time for completion of translation projects involving PerfO measures relative to PRO measures?
   1. PerfOs usually take more time to complete relative to PRO measures
   2. PerfOs usually take the same amount of time to complete as PRO measures
   3. PerfOs usually take less time to complete relative to PRO measures

**Translation and Linguistic Validation Process**

1. What types of differences do you find in the amount of explanation provided for correctly rendering concepts that are being measured in PerfOs relative to PRO measures?
   1. Usually more conceptual explanation or elaboration is provided over PRO measures
   2. No differences noted between conceptual explanations provided for PerfO measures and PRO measures
   3. Less conceptual explanation is usually provided for PerfO measures compared to PRO measures
2. What steps do you feel should be included in the translation of PerfO measures? (mark all that apply):
   1. Creation of concept definition document
   2. Developer review of concept definition document
   3. Single forward translation
   4. Dual forward translation
   5. Reconciliation of forward translations
   6. Single back translation
   7. Dual back translation
   8. Project Manager review and evaluation of back translations
   9. Developer review of back translation
   10. In country (in specialty) clinician review of translation (single clinician reviewer)
   11. In country (in specialty) clinician review of translation (multiple clinicians)
   12. Client subsidiary review of translation (i.e. in-country review by a study physician or by the on-site field coordinator)
   13. Pilot-testing of the tasks by patients in country, where required activities are performed
   14. Patient review (cognitive interview process) of patient facing aspects of a PerfO measure (e.g., instructions to patient)
   15. Developer review of cognitive interview results
   16. Proofreading
   17. Other - Please specify and describe:­­­­­­­­­­­­­­­­­­­­____________________________________________________
3. When completing translations of a PerfO measure, what type of input from clinician(s) do you typically plan:
   1. Review of the translated material by clinician(s) – clinician(s) may propose(s) changes to translation but without direct discussion with project team
   2. Cognitive interview process with clinician(s) - specific questions asked to clinician(s) during interview about translated PerfO measure
   3. Another type of input from clinician(s), please describe: ____________________________________________________
   4. No clinician’s input planned
4. When completing translations of a PerfO measure, do you typically plan input from clinician(s):
   1. At one point in the process
   2. At several points in the process. If so, please describe how many and when in the process: ____________________________________________________
   3. No clinician’s input planned
5. What is the general level of acceptable credentials for clinician(s) involved in the process for PerfO measures?
   1. Not applicable: no clinician’s input planned
6. Are there any differences in the guidance you provide to linguists / reviewers regarding best practices for translation and linguistic validation when working with PerfO measures vs. PRO measures?
   1. Yes
   2. No

Please explain your answer in a sentence or two:

1. Who should conduct the translation process for a PerfO measure? (mark all that apply)
   1. Translation consultant (non-medical)
   2. Medical translator
   3. Clinical care professionals
   4. Other? (specify) _______________________________________

**Recruitment, Pilot Testing and Cognitive Interview Differences**

1. When completing translations of a PerfO measure, do you typically plan pilot testing, i.e. administering the PerfO measure where respondents will perform the tasks?
   1. Yes
   2. No

If yes, who administers the PerfO measure:

1. The interviewer(s) who will then be in charge of cognitive interviews. If yes, how many interviewer(s) typically:___________
2. A different person experienced in administration & scoring of similar PerfO measures. If yes, how many typically: _____________
3. Someone else

If someone else, please specify: ___________________________________________

1. When completing translations of a PerfO measure, do you typically plan cognitive interviews with respondents, where patient-facing parts (e.g. instructions, stimuli) are reviewed with respondents?
   1. Yes
   2. No
2. When completing translations of a PerfO measure, do you typically plan cognitive interviews with the person who administered the PerfO measure during pilot testing, where instructions to rater are reviewed?
   1. Yes
   2. No
3. When completing cognitive interviews of translated PerfO measures, please select all of the process steps you would generally include:
4. Cognitive interviews for clinician-facing parts with clinicians/healthcare professionals. If yes, specify number of interviews:
5. Cognitive interviews for patient-facing parts with clinicians. If yes, specify number of interviews:
6. Cognitive interviews with patients for patient-facing parts. If yes, number of interviews:
7. Cognitive interviews with “healthy” subjects (those without a particular disease or illness) for patient-facing parts. If yes, number of interviews:
8. Review of cognitive interview methodology by developer or sponsor prior to interviews
9. Review of cognitive interview data by developer or sponsor prior to translation delivery
10. Other(s), please describe: _________________________________________________
11. When the PerfO measure is planned to be used with cognitively impaired patients, what type of population do you typically conduct pilot testing and cognitive interviews with?
    1. Cognitively impaired patients
    2. Cognitively intact patients
    3. Cognitively intact “healthy” subjects (those without a particular disease or illness) **without** other specific criteria
    4. Cognitively intact “healthy” subjects (those without a particular disease or illness) **with** other specific criteria e.g. within a specific age range
    5. Other(s), please describe: _________________________________________________

**Developer Involvement**

1. Are there any differences in the level of client or developer involvement in a project with PerfO translation compared to PROs?
   1. **More** developer involvement relative to PROs
   2. **Equal** levels of developer involvement relative to PROs
   3. **Less** developer involvement relative to PROs

**General**

1. Are there any other special considerations you would recommend when planning translation & linguistic validation of PerfO measures?
   1. No
   2. Yes

If yes, please describe: _________________________________________________________

1. If there are any other comments you would like to contribute relative to the translation process for PerfO measures, please write those comments here.
2. Thank you for your participation. Please provide the name of the company or organization you represent below:
